# Upper extremity vein thrombosis: an alert symptom of breast cancer in elderly patients. Experience on personal casuistry and review of the literature

**DOI:** 10.1186/1471-2482-13-S2-S39

**Published:** 2013-10-08

**Authors:** Raffaele Serra, Rita Compagna, Raffaele Grande, Paolo Perri, Gianluca Buffone, Vincenzo Gasbarro, Antonello Accurso, Michele Danzi, Giovanni Aprea, Bruno Amato, Stefano de Franciscis

**Affiliations:** 1 Interuniversity Center of Phlebolymphology - Headquorters at University Magna Graecia of Catanzaro - Viale Europa, Località Germaneto - 88100 Catanzaro, Italy; 2 Department of Medical and Surgical Science-University Magna Gracia of Catanzaro - Viale Europa, Località Germaneto - 88100 Catanzaro, Italy; 3 Department of Surgery - University of Ferrara, 44100, Ferrara, Italy; 4 Department of General, Geriatric, Oncologic Surgery and Advanced Technologies, University "Federico II" of Naples. Via Pansini, 5 - 80131 - Naples, Italy

## Abstract

**Background:**

Breast Cancer in elderly patients is a significant health problem representing an important source of morbidity and mortality. Although the most common presentation is the presence of a palpable lump there may be, especially in the elderly population, rare clinical manifestations such as thromboembolic events that often involve the upper limbs.

**Methods:**

We retrospectively reviewed a ten year clinical casuistry of patients with Breast Cancer who presented for an initial diagnosis of upper extremity vein thrombosis.

**Results:**

13 patients with initial diagnosis of upper limbs vein thrombosis (1M-12 F; age range 48-76; median age 70 years) resulted affected from Breast Cancer. The diagnosis of vein thrombosis represented the first clinical manifestation related to thier malingancy. All patients of our casuistry had positive ER/PR receptor status.

**Conclusions:**

A case of upper vein extrmity thrombosis in an elderly patient should prompt a high index of suspicion for breast malignancy in order to avoid diagnostic delay that may retard appropriate treatment.

## Background

Although the number of elderly patients with breast cancer (BC) is increasing, knowledge regarding clinical characteristics and biology of this disease in old age is limited [1].

BC and Venous Thromboembolism (VTE) are two conditions often correlated more than expected [2-6].

Some studies demonstrated a higher number of ER/PR expression in older patients [1] and another recent retrospective cohort ctudy showed that clinical manifestations related to alterations of circulatory venous system are highly prevalent in patients with BC with positive ER and/or PR status [3].

Aim of this study is to review our clinical casuistry of patients with BC who presented at our clinical departments with initial diagnosis of upper extremities vein thrombosis.

### Methods

After obtaining institutional review board approval, we retrospectively reviewed the charts of 13 patients (1M-12F; age range 48-76; median age 70 years) presented with a new breast cancer diagnosis together with a concomitant upper extremity vein thrombosis. The casuistry belonged to three Clinical Departments of the Interuniversity Center of Phlebolymphology.

Demographics and clnical characteristics are reported in Table 1.

**Table 1:** Baseline Patients Characteristics.

Population study		%
**Number of patients**	13	100

**Sex (M/F)**	1/12	7.69%/92.31%

**Age** Range 48-76 median age 70 48-65 66-70 70 +	2 6 5	15.38% 46.15% 38.46%

**BC stage** Stage I Stage II Stage III Stage IV	1 3 5 4	7.69% 23.08% 38.46% 30.76%

**BC Histology** Ductal Lobular	9 4	69.23% 30.77%

**ER/PR status** ER+/PR+ ER+/PR- ER-/PR+ ER-/PR-	10 2 0 0	76.92% 15.38% 0% 0%

**Vein Thrombosis** Subclavian Vein Axillary Vein Brachial Vein Cephalic Vein Side: right left	5 5 2 1 7 6	38.46 38.46 15.38% 7.69% 53.85% 46.15%

### Results and discussion

In our casuistry, in a 10 years period, 13 patients presented with a several week history of painful upper extremities edema.

An initial diagnosis of upper extremity vein thrombosis was made. Subclavian/axillary veins were involved in 76.92%, Brachial vein in 15.38% and Cephalic vein in 7.69% of cases.

All patients denied past history of vascular thrombosis, pulmonary embolism or recent surgery on the affected arm. Breast examination revelead symmetrical breast without skin dimpling or nipple retraction except in one case previously described in which a small area with a slight dimpling of the skin and formation of wrinkles was noted [4].

All patients were examined for a possible breast malignancy and dignosis of BC was confirmed clinically and instrumentally as described in Tab. I.

All patients underwent anticoagulant therapy with low molecular weight heparin for vein thrombosis and subsequent adapted surgical and/or medical treatment for their cancer conditions as previosuly described [2-4].

All patients of our casuistry had positive ER/PR receptor status and this accounts for a more favourable biological characteristic (low proliferative rates, diploidy, normal p53) and this allowed also to use hormonal treatment as it is the first choice medical treatment in the majoirity of elderly breast cancer [7,8].

Four patients (30.76%) of our casuistry had a moderate clinical stage (I-II) while 9 patients (69.22) had a more severe clinical stage (III-IV).

Data from scientific literature shows that 48% of breast cancer cases occur in patients aged 65 and older, and more than 30% occur in those over the age of 70 [8,9].

BC, due to its indolence in elderly patients, is frequently diagnosed at a more advanced stage and in these cases local and systemic treatments are often less effective.

Although the most common presentations of breast cancer are a palpable mass, there are less common clinical features that must be considered, especially in the elderly, as thromboembolic events often involving the upper limbs.

VTE and cancer have a two-way clinical association: VTE may be the presenting symptom of an occult cancer or, on the other side, patients with clinically overt malignancy may develop VTE as a complication at any stage of their disease [5].

## Conclusions

Atypical clinical presentations, such as thromboembolic events, especially involving the upper limbs, are often the first clinical symptoms of BC in elderly patients, and if promptly recognized, may allow an early diagnosis of malignancy in these kind of patients.

## List of abbreviations

BC: Breast Cancer; ER+: estrogen receptor - positive status; ER-: estrogen receptor - negative status; PR+: progesterone receptor - positive status; PR-: progesterone receptor - negative status; VTE: Venous Thromboembolism;.

## Competing interests

The authors declare that they have no competing interests.

## Authors' contributions

RS: conception and design, interpetration of data, given final approval of the version to be published. RC: acquisition of data, drafting the manuscript, given final approval of the version to be published. RG: acquisition of data, drafting the manuscript, given final approval of the version to be published. PP: acquisition of data, drafting the manuscript, given final approval of the version to be published. GB: critical revision, interpretation of data, given final approval of the version to be published. VG: critical revision, interpretation of data, given final approval of the version to be published. AA: acquisition of data, drafting the manuscript, given final approval of the version to be published. MD: acquisition of data, drafting the manuscript, given final approval of the version to be published. GA: acquisition of data, drafting the manuscript, given final approval of the version to be published. BA: critical revision, interpretation of data, given final approval of the version to be published. SdF: conception and design, critical revision, given final approval of the version to be published.

## Authors' information

RS: Assistant Professor of Surgery at University Magna Graecia of Catanzaro, Head Master Training Programme in Wound Care, Vascular Surgeon at University Hospital of Catanzaro. RC: Post-Graduate Doctorate in Vascular Surgery at University "Federico II" of Naples. RG: Student School of Medicine at University Magna Graecia of Catanzaro. PP: Resident in Vascular Surgery Training Programme at University Magna Graecia of Catanzaro. GB: Research Fellow at University Magna Graecia of Catanzaro. VG: Associate Professor at University of Ferrara. AA: Aggregate Professor of Surgery at Naples University Federico II. MD: Aggregate Professor of Surgery at Naples University Federico II. GA: Aggregate Professor of Surgery at Naples University Federico II. BA: Associate Professor of Surgery at University "Federico II" of Naples. SdF: Full Professor of Surgery at University Magna Graecia of Catanzaro, Head of Vascular Surgery Training Programme at University Magna Graecia of Catanzaro, Head of PhD Programme in Phlebolymphology Clinical and Experimental, Chief Division of Vascular Surgery at University Hospital of Catanzaro.

## Declarations

Publication of this article was funded by author's personal funds.

This article has been published as part of *BMC Surgery *Volume 13 Supplement 2, 2013: Proceedings from the 26th National Congress of the Italian Society of Geriatric Surgery. The full contents of the supplement are available online at http://www.biomedcentral.com/bmcsurg/supplements/13/S2

## References

[B1] Pappo I Karni T Sandbank J Dinur I Sella A Stahl-Kent V Wasserman I Halevy A Breast cancer in the elderly: histological, hormonal and surgical characteristics Breast 2007 13 1 60 7 10.1016/j.breast.2006.05.007 17276293

[B2] Serra R Buffone G Montemurro R de Franciscis S Axillary vein thrombosis as the first clinical manifestation of inflammatory breast cancer: report of a case Surg Today 2013 13 1 100 2 10.1007/s00595-012-0196-7 22618999

[B3] Serra R Buffone G Miglietta AM Abonante S Giordano V Renne M Lugarà M de Franciscis S Breast Cancer and Venous Disease: A Retrospective Cohort Study Ann Vasc Surg 2013 13 6 762 766 10.1016/j.avsg.2012.10.020 23809843

[B4] Serra R Buffone G Perri P Renne M Amato B de Franciscis S Male breast cancer manifesting as Cephalic Vein Thrombosis in a 70-year-old patient Ann Vasc Surg 2013 in press (doi 10.1016/j.avsg.2013.01.016.) 10.1016/j.avsg.2013.01.016 23988541

[B5] Kruger SJ Breast Cancer presenting as subclavian/axillary deep vein thrombosis and upper limb lymphoedema Ann R Coll Surg Eng 2012 13 55 56 10.1308/003588412X13171221500745 PMC5827237 22391349

[B6] Amato B Rispoli C Iannone L Testa S Compagna R Rocco N Surgical margins of resection for breast cancer: Current evidence Minerva Chirurgica 2012 13 5 445 452 23232484

[B7] Rispoli C Rocco N Iannone L Compagna R Cacciapuoti MT Bellino A Amato B Breast reconstruction in older women: A growing request BMC Geriatrics 2009 13 SUPPL. 1 A46

[B8] Wildiers H Paridaens R Taxanes in elderly breast cancer patients Cancer Treat Rev 2004 13 4 333 42 10.1016/j.ctrv.2003.12.001 15145508

[B9] Rispoli C Rocco N Iannone I Amato B Developing guidelines in geriatric surgery:role of the grade system BMC Geriatrics 2009 13 SUPPL 1 article n.A99

